# Up-regulated microRNA-33b inhibits epithelial–mesenchymal transition in gallbladder cancer through down-regulating CROCC

**DOI:** 10.1042/BSR20190108

**Published:** 2020-01-10

**Authors:** Guohui Xu, Xiaoyong Wei, Qiang Tu, Cuncai Zhou

**Affiliations:** Department of Hepatobiliary Tumor Surgery, Jiangxi Cancer Hospital, Nanchang 330029, P. R. China

**Keywords:** CROCC, Epithelial-mesenchymal transition, Gallbladder cancer, Invasion, microRNA-33b, Proliferation

## Abstract

Gallbladder cancer (GBC) is a relatively rare but fatal gastrointestinal tumor. The microRNA-33b (miR-33b), a member of miR-33 family, is reported to function as a tumor suppressor in various cancers. Notably, miR-33 was predicted to target CROCC based on microarray-based analysis. Hereby, we aimed to characterize the effect of miR-33b on epithelial–mesenchymal transition (EMT) in GBC and the potential mechanism involved with the regulation of CROCC. In GBC cell lines, miR-33b expressed at low levels, and CROCC expressed at high levels, with enhanced EMT process. To further examine the specific mechanism of miR-33b and CROCC in GBC, the GBC cells were treated with the miR-33b mimic/inhibitor or siRNA-CROCC to assess the expression alteration of EMT-related genes and cell proliferation, migration, and invasion. MiR-33b was verified to target and down-regulate the expression of CROCC. The miR-33b up-regulation or CROCC silencing was observed to increase the level of E-cadherin but decrease the levels of N-cadherin and Vimentin, corresponding to impeded cell proliferation, migration, invasion, EMT, and tumor growth. The findings suggest that miR-33b up-regulation hinders GBC development through down-regulating CROCC, which was achieved by inhibition of EMT. The present study may provide an insight on a novel target for GBC treatment.

## Introduction

Gallbladder cancer (GBC) is the most prevalent type of metastatic carcinoma in the biliary tract, accounting for 80–95% of clinically diagnosed biliary tract cancers worldwide and is acknowledged as the sixth most common type of cancer among gastrointestinal cancers [1]. With comprehensive advancements in diagnosis and therapy, the 5-year survival rate for GBC has been persistently low, at about 13–30% [2]. For patients with incidental GBC, the average survival time is 26.5 months, with a shorter overall survival of about 9.2 months for patients with suspected GBC [3]. The major risk factors for GBC incorporate gallstones, progressing age, female gender, anomalous pancreaticobiliary ductal junction, certain ethnic groups, and geographic influence [4]. In spite of the advancements in treatment modalities for GBC such as surgical intervention, radiotherapy, and chemotherapy over the decades, the 5-year survival rate of patients with GBC remains low [5]. Thus, it is pivotal to investigate the molecular mechanisms of GBC to improve treatment modalities and prolong the survival time of GBC patients [6].

Reports have illustrated the functionality of microRNAs (miRs) in diverse biological and cellular development of many severe diseases including GBC [7]. For instance, miR-26a was poorly expressed in GBC, and hence it could serve as a potential marker for early prognosis and treatment of GBC patients [8]. Furthermore, miR-33b, which belongs to the miR-33 family, is an intronic miR encoded within the sterol response element binding protein 1 (SERBP-1) gene [9]. Particularly, up-regulated miR-33b has been reported to exert its tumor suppressive properties for inhibiting lung squamous cell carcinoma cell proliferation and migration *in vitro* [10]. In addition, miR-33b was also found to be down-regulated in primary tumor samples and osteosarcoma cell lines, which flagged the potential of an overexpression of miR-33b to inhibit cell proliferation, migration, and invasion in osteosarcoma [11]. Additionally, the biological prediction by the RNA22 database demonstrated the ability of miR-33b to specifically bind to the ciliary rootlet coiled coil protein (CROCC), which was also ascertained in our experimentation. CROCC is also known as ROLT or TAX1 Binding Protein 2 (TAX1BP2) [12]. Tax is a transcriptional activator, which evidently influences cell signaling through modulation of the CRE, κB, and SRE pathways and on the expression of various cytokines and proto-oncogenes, which leads to excessive centrosome duplication by targeting a specific centrosomal protein, TAX1BP2 [13]. Reports have flagged the functionality of CROCC with vital roles in tumors and involvement in the expression of cytokines and cancer-related genes. The aforementioned evidences led to a hypothesis that miR-33b and CROCC may be potentially involved in the development of GBC. Therefore, the present study was planned to explore the effect of miR-33b on GBC and its mechanism involving CROCC.

## Materials and methods

### Dual luciferase reporter gene assay

The GBC-related miRNA microarray database GSE104165 was retrieved from the Gene Expression Omnibus (GEO) database (https://www.ncbi.nlm.nih.gov/) and the database with GBC tissues (*n* = 40) and adjacent normal tissues (*n* = 8) were then subjected to differential expression analysis with |log2FC| > 1, *P* value < 0.05 as threshold. Next, a volcano plot of differentially expressed genes was plotted. The target gene of miR-33b was analyzed using the RNA22 database (https://cm.jefferson.edu/rna22), after which dual luciferase reporter gene assay was performed to verify whether CROCC was a direct target gene of miR-33b. The CROCC 3′ untranslated region (3′UTR) gene fragments were synthesized and introduced to the pGL 3-control (Promega Corporation, Madison, WI, U.S.A.) using the endonuclease sites XhoI and BamHI, respectively. Complementary sequence mutation site of the seed sequence was designed on the wide type (WT) CROCC. The target fragment was inserted into the pGL3-control vector using T4 DNA ligase after utilizing restrictive endonuclease. The sequence confirmed that the luciferase reporter plasmids WT and mutant type (MUT) were co-transfected with the miR-33b mimic respectively into HEK-293T cells (Shanghai Institute of Life Sciences, Shanghai Academy of Sciences Cell Resource Center, Shanghai, China). After 48 h, the cells were collected and lysed. Next, the dual luciferase reporter assay system kit (Promega, U.S.A.) was employed to detect the luciferase activity of HEK-293T cells using a Luminometer TD-20/20 detector (E5311, Promega, U.S.A.). Each experiment was repeated three times independently.

### Cell culture

Human gallbladder epithelial cells HGBEC and GBC epithelial cells SGC-996 were acquired from Tongji University Medical School Cancer Cell Research Center, and the GBC cell line NOZ was bought from Japan Health Research Resource Bank (HSRRB). The GBC cell line GBC-SD was acquired from the Shanghai Institute of Cellular Sciences of Chinese Academy of Sciences and the GBC cell line QBC939 was acquired from Shanghai FuHeng Biology Co., Ltd (Shanghai, China). All cell lines were cultured in Dulbecco’s modified Eagle medium (DMEM, Gibco, New York, U.S.A.) with 10% of fetal bovine serum (FBS) (Hangzhou Lookchem Biologyl Engineering Material Co., Ltd., Hangzhou, Zhejiang, China) and 1% of double antibody (100 U/l of penicillin and 100 mg/l of streptomycin, Gibco, New York state, U.S.A.) at 37°C with 5% CO_2_. These cell lines were sub-cultured once every 2–3 days and cell lines in the logarithmic growth stage were selected for further experimentation. Reverse transcription quantitative polymerase chain reaction (RT-qPCR) was applied to clarify the expression of miR-33b in different GBC cell lines.

### Cell sub-culture and cell treatment

Cells were treated with 0.25% trypsin after sub-culture using DMEM with 10% FBS in a ratio of 1:2 or 1:3, and then the cells were incubated at 37°C with 5% CO_2_. The sub-culture was conducted every 2 days on observing cell fusion. When cells were treated with 0.25% trypsin for 30 s, the culture medium was collected and centrifuged at 179 × *g* for 2 min with the supernatant discarded. Subsequently, 1-ml fresh complete medium at room temperature was used to re-suspend the cells, after which the cells were mixed and stored into two clean and sterile culture flasks in the ratio of 1:2 and 1:5, respectively. Next, the complete medium was complemented to 4 ml in each culture flask and incubated at 37°C with 5% CO_2_. Cells in the logarithmic growth period were selected for further experimentation. The GBC cells were assigned into seven groups: the mimic NC group, the miR-33b mimic group, the inhibitor NC group, the miR-33b inhibitor group, the inhibitor NC + si-NC group, the inhibitor NC + si-CROCC group, and the miR-33b inhibitor + si-CROCC group, respectively. The cells were seeded in a 6-well plate for 24 h before transfection. Upon attaining 50% cell confluence, the human GBC cells were transfected according to lipofectamine 2000 (Invitrogen, U.S.A.). After 6 h, the culture medium was changed. Cells were extracted for follow-up experiment after culturing for 48 h.

### Immunofluorescence staining

The localization and expression of EMT-related markers (E-cadherin, N-cadherin, and Vimentin) were detected in strict accordance with the provided instructions of the immunofluorescent staining kit (15251, Shanghai Active Motif Biotech Co., Ltd, Shanghai, China). The steps were as follows: the slides of GBC cells were prepared in a 24-well plate, which were subjected to a rinse with phosphate-buffered saline (PBS) and finally fixed using stationary liquid. Next, immunostaining-washing solution was used twice on the shaker (each time for 5 min). Next, immunostaining-blocking solution was applied to block the cells for 60 min. Subsequently, immunostaining primary antibody diluent was added to dilute E-Cadherin (1:3000; ab15148; Abcam Inc., Cambridge, MA, U.S.A.), N-Cadherin (1:1000; ab18203; Abcam Inc., Cambridge, MA, U.S.A.), Vimentin (1:1000; ab45939; Abcam Inc., Cambridge, MA, U.S.A.), followed by incubation at 4°C overnight. Then the secondary antibody (Abcam, U.K., ab6717, goat anti-rabbit, 1/1000-1/5000) labeled by fluorescein isothiocyanate was diluted using an immunostaining secondary antibody diluent and further incubated at room temperature for 1 h. The nucleus was stained using 4′,6-diamidino-2-phenylindole (DAPI) for 5 min, and the slices were sealed immediately. The levels of several EMT-related markers (E-cadherin, N-cadherin, and Vimentin) in each group were observed under a fluorescence microscope (Nikon Corporation, Tokyo, Japan).

### RT-qPCR

RNA extraction kit (Invitrogen Inc., Carlsbad, CA, U.S.A.) was utilized to extract the total RNA of GBC cells in each group. The primers (Table 1) were designed and then synthesized by the Takara company (Kyoto, Japan). Next, the PrimeScript RT kit was utilized to reversely transcribe RNA into cDNA in strict accordance with the provided instructions of the kit. ABI PRISM® 7300 was used to conduct RT-qPCR. U6 was served as an internal control for the relative level of miR-33b, while glyceraldehyde-3-phosphate dehydrogenase (GAPDH) served as an internal control for the genes of interest. The 2^−△△*C*^_T_ method was adopted to calculate the relative transcription levels of the respective genes [14].

**Table 1:** Primer sequences of RT-qPCR

Gene	Primer sequence (5′ - 3′)
miR-33b	F: 5′-TGTCAGGCAACCGTATTCACC-3′
	R: 5′-CATGCAGTGAGTTAGATGTAGAACGTGCATTGCTGT-3′
CROCC	F: 5′-GGTGGAGCTGACACTAGAGAC-3′
	R: 5′-GGCTGGTGATCTGAGCGTC-3′
E-cadherin	F: 5′-GTAACCGATCAGAATGAC-3′
	R: 5′-CGTGGTGGGATTGAAGAT-3′
N-cadherin	F: 5′-AGTCAACTGCAACCGTGTGT-3′
	R: 5′-AGCGTTCCTGTTCCACTCAT-3′
E-cadherin	F: 5′-GGAGCAGAAAGCAGAACCC-3′
	R: 5′-TTCCTTCCACGAAACCAGTG-3′
Vimentin	F: 5′-AAATGGCTCGTCACCTTGG-3′
	R: 5′-TGGGTATCAACCAGAGGGAGT-3′
U6	F: 5′-ATACAGAGAAAGTTAGCACGG-3′
	R: 5′-GGAATGCTTCAAAGAGTTGTG-3′
GAPDH	F: 5′-GGAGCGAGATCCCTCCAAAAT-3′
	R: 5′-GGCTGTTGTCATACTTCTCATGG-3′

Notes: F, forward; R, reverse; miR-33b, microRNA-33b; RT-qPCR, reverse transcription quantitative polymerase chain reaction; GAPDH, glyceraldehyde-3-phosphate dehydrogenase.

### Western blot analysis

The total protein was extracted from the cultured cells in each group and the tumor tissues of nude mice. The 5× sodium dodecyl sulfate (SDS) loading buffer was added, following by electrophoretic separation with polyacrylamide gel electrophoresis (SDS-PAGE). Next, the proteins were transferred onto a polyvinylidene fluoride membrane, which was then blocked. After washing with tris-buffered saline tween (TBST), the membrane was respectively incubated at 4°C overnight with the primary antibodies of CROCC (NBP1-80820; 1: 200; Bio-Techne, China), E-Cadherin (1: 3000; ab15148; Abcam Inc., Cambridge, MA, U.S.A.), N-Cadherin (1: 1000; ab18203; Abcam Inc., Cambridge, MA, U.S.A.), Vimentin (1: 1000; ab45939; Abcam Inc., Cambridge, MA, U.S.A.), and β-actin (1:1000, ab8226, Abcam Inc., Cambridge, MA, U.S.A). After a rinse with TBST, the membrane was incubated with horseradish peroxidase (HRP)-labeled secondary antibody at room temperature for 1 h. Next, the proteins were visualized by HRP-labeled enhanced chemiluminescence, with documentation of the images. The grey value of target band was analyzed using the ImageJ software.

### 5-ethynyl-2′-deoxyuridine (EdU) incorporation

The transfected GBC cells in the logarithmic phase were selected. Next, 250 μl of working solution (a final concentration of 10 μl) was added to a 24-well cell culture plate (250 μl). Forty-five minutes after incubation, the culture solution was removed, and the plate was rinsed using 1× PBS three times, followed by the addition of 500 μl of 3.7% formaldehyde/PBS for fixation at room temperature for 15 min. Subsequently, the fixing solution was removed, and the plate was rinsed with 500 μl of 3% bovine serum albumin (BSA)/PBS twice. Next, 250 μl of 0.5% TritonX-100/PBS per well was added to the plates and then incubated at room temperature for 20 min. After that, 10× stock solution was diluted into 1× Click-iT EdU buffer additives by the addition of ddH_2_O for preparation of the Click-iT reaction mixture (best to use within 15 min after preparation) after which the permeabilizing solution was removed. The 500 μl of 3% BSA/PBS was used to rinse the plate twice and 500 μl of Click-iT reaction mixture was added into each well. Next, the cells were subjected to incubation for 30 min at room temperature (avoiding exposure to light). Subsequently the Click-iT reaction mixture was removed, and the cells were rinsed again with 500 μl of 3% BSA/PBS. Mounting medium containing DAPI was utilized to seal the cell slides. The EdU incorporation of cells was observed and analyzed under a fluorescence microscope.

### Transwell assay

The cell migration experiment was conducted using transwell chamber (0.8 μm pore) (Corning Glass Works, Corning, N.Y., U.S.A.), and the Matrigel (0.8 μm pore) was used for transwell invasion. The third generation of cells was selected to grow in the chamber until the cell confluence reached 80%. The cells were starved by culturing with serum-free DMEM for 24 h. Serum-free DMEM (Corning, Corning, New York, U.S.A.) was added to the basolateral chamber and placed at room temperature for 1 h. After that, the cells were re-suspended using serum-free DMEM after digestion. Next, the cells were counted and diluted to 3 × 10^5^ cells/ml. Next, 100 μl of cells and 600 μl of 10% DMEM containing 10% serum (serum as chemokine) were introduced in the apical chamber. The chamber was incubated for 24 h based on the provided instructions of the transwell chambers. The cells in the apical chamber were removed. Next, the apical chamber was rinsed with PBS and soaked with pre-cooled methanol for 30 min. The cells transferred to the basolateral chamber were fixed and stained using 0.1% Crystal Violet solution for 10 min. Six fields were selected and an Olympus inverted microscope (Olympus, Japan) were used to take photos and count cells. The experiment was repeated three times independently. In the cell invasion experiment, Matrigel was acquired from a −20°C freezer and melted in a 4°C fridge. Serum-free DMEM was diluted according the proportion of 1:10 for further use. The concentration of inoculation cells was adjusted to 1.0 × 10^5^ cells/ml and other operation followed a similar protocol as the migration experiment. The experiment was performed three times independently. The rate of migration and invasion = the migration and invasion cells/total cells.

### Tumor formation in nude mice

Forty-two nude mice at the age of 4–5 weeks old were purchased from SLAC Laboratory Animal Co. Ltd (Shanghai, China). The mice were then fed in individual cages in a specific pathogen free (SPF) animal laboratory with the humidity of 60–65%, and the temperature of 22–25°C. The mice were accessed to free food and water under a 12-h light/dark cycle. The experiment was conducted after one-week adaptive feeding. The health state of nude mice was observed prior to the experiment. Stably transfected GBC cells in the logarithmic growth phase were obtained. The nude mice were anesthetized by pentobarbital sodium (30 mg/kg) and subcutaneously inoculated with 2 × 10^7^ cells/ml cell suspension (100 μl for each mice) in the axilla of the hind limbs. The nude mice were classified into seven groups (*n* = 6): the mimic NC group, the miR-33b mimic group, the inhibitor NC group, the miR-33b inhibitor group, the inhibitor NC + si-NC group, the inhibitor NC + si-CROCC group, and the miR-33b inhibitor + si-CROCC group. The maximum diameter (*L*) and minimum diameter (*W*) of tumor weight were measured weekly using a vernier caliper. The size of the tumor was calculated using *V* = *W*^2^ × *L* × 0.52 [15]. On the 35th day, the nude mice were killed by carbon dioxide asphyxiation and the tumors were excised. The expression of CROCC and EMT-related proteins was detected using Western blot analysis.

### Statistical analysis

SPSS 21.0 statistical software (IBM Corp. Armonk, NY, U.S.A.) was adopted for data analysis. Each experiment was repeated at least three times for estimation of the mean and standard deviation. Measurement data were represented by mean ± standard deviation. With normal distribution and homogeneity of variance, the data between two groups were analyzed by *t* test, and data between multiple groups were compared by one-way analysis of variance (ANOVA), followed by pairwise comparisons as post hoc test. Otherwise, the data between two groups were analyzed using the Wilcoxon rank sum test and data between multiple groups were analyzed using Cruskal–Wallis rank sum test. Data at different time points were assessed using repeated measures ANOVA. A value of *P* < 0.05 was considered to be statistically significant.

## Results

### miR-33b expression is down-regulated in GBC cell lines

**Figure 1:**
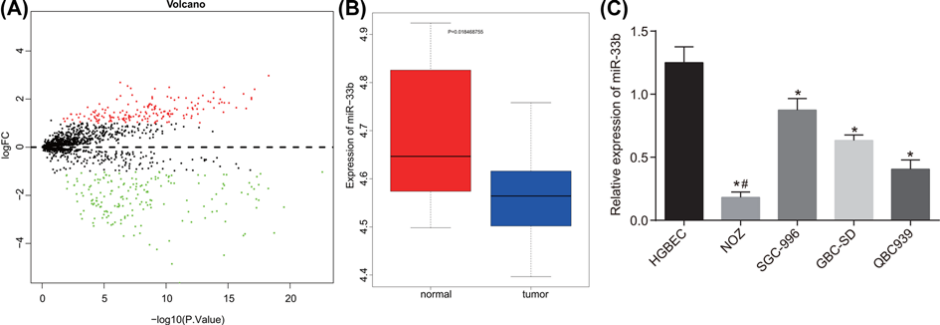
miR-33b is poorly expressed in GBC cell lines (**A**) Expression volcano plot of genes retrieved from the GSE104165 microarray database. (**B**) Box plot of miR-33b expression in GBC tissues and adjacent normal tissues in the GSE104165 microarray database. (**C**) miR-33b expression in GBC cell lines; *, *P* < 0.05 vs. normal gallbladder epithelial cell line; #, *P* < 0.05 vs. the rest GBC cell lines; the data in the figure were analyzed by one-way ANOVA; the experiment was repeated three times independently; miR-33b, microRNA-33b; RT-qPCR, reverse transcription quantitative polymerase chain reaction; GBC, gallbladder carcinoma; NC, negative control.

The GBC-related miRNA microarray database GSE104165 was retrieved from the Gene Expression Omnibus (GEO) database. Then, a volcano plot of the screened differentially expressed genes was plotted. As shown in Figure 1A,B, miR-33b presented with a significantly differential expression in GBC with a poor expression. RT-qPCR was performed in order to detect the level of miR-33b in the normal gallbladder epithelial cell line (HGBEC) and the four GBC cell lines (QBC939, SGC-996, NOZ, and GBC-SD). The results (Figure 1C) indicated that miR-33b presented with low levels in all four GBC cell lines compared with those in the normal gallbladder epithelial cells. The expression of miR-33b varied in these four cell lines, specifically in the NOZ as it was significantly lower compared with the other cell lines (*P* < 0.05). GBC cell line NOZ was selected for further experimentation.

### MiR-33b targets and down-regulates the CROCC expression in GBC

**Figure 2:**
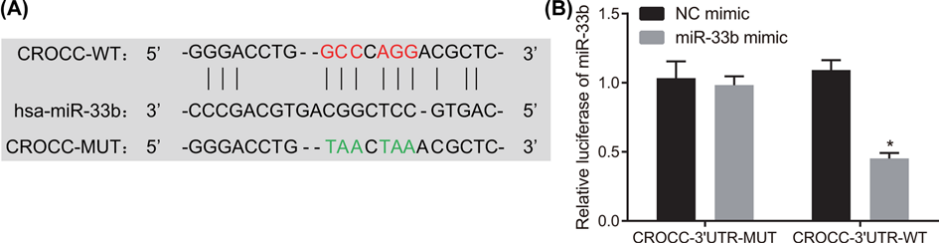
MiR-33b targets and down-regulates the CROCC expression in GBC (**A**) The binding sites predicted by RNA22 database (https://cm.jefferson.edu/rna22) between miR-33b and CROCC gene. (**B**) Luciferase activity of CROCC-3′UTR in the NC mimic and miR-33b mimic groups detected by dual luciferase reporter gene assay. The data in panel (B) were analyzed using the independent sample *t* test; the experiment was repeated three times independently; wt, wild-type; mut, mutant; miR-33b, microRNA-33b; NC, negative control.

The RNA22 database (https://cm.jefferson.edu/rna22) was applied to predict the target gene of miR-33b, and the results revealed the presence of a specific binding region between the CROCC gene sequence and the miR-33b sequence (Figure 2A). Additionally, CROCC (TAX1BP2) has been reported to function as a tumor suppressor in various cancers [16,17]; however, the role of CROCC in GBC was scarcely reported. CROCC was verified as a target gene of miR-33b by dual luciferase report gene assay. The results demonstrated (Figure 2B) that compared with NC mimic treatment, miR-33b mimic treatment resulted in decreased luciferase activity in the CROCC-3′UTR-Wt group (*P <* 0.05), but it did not influence the luciferase activity in the CROCC-3′UTR-MUT group (*P* > 0.05), which suggested that miR-33b could extensively target the CROCC gene and subsequently down-regulate its expression.

### MiR-33b down-regulates the expression of CROCC to suppress proliferation in GBC cells

**Figure 3:**
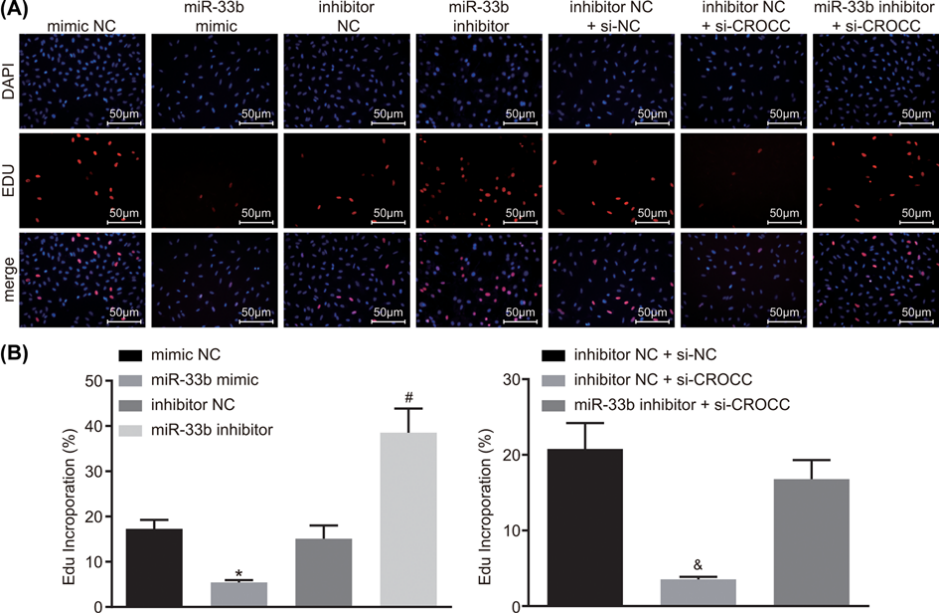
MiR-33b down-regulates the expression of CROCC to attenuate the proliferation of GBC cells (**A**) Representative images of EdU staining for cells in each group. (**B**) The EdU-positive GBC cells in each group; *, *P* < 0.05 vs. the mimic NC group; #, vs. the inhibitor NC group; &, vs. the inhibitor NC + si-NC group; the data in the figure were analyzed by one-way ANOVA, and the experiment was repeated three times independently; EdU, 5-ethynyl-2-deoxyuridine; miR-33b, microRNA-33b, NC, negative control, GBC, gallbladder carcinoma.

EdU detection was performed in order to evaluate the positive expression rate of cells in each group. The results (Figure 3A,B) showed that compared with the mimic NC group, the EdU positive expression rate of cells in the miR-33b mimic group significantly decreased (*P* < 0.05), which implied that cell viability was inhibited. In comparison with the inhibitor NC group, the miR-33b inhibitor group presented with an evidently elevated EdU positive expression rate of cells (*P* < 0.05), suggesting for increased cell viability. A comparison with the inhibitor NC + si-NC group demonstrated a significantly declined EdU positive expression rate of cells in the inhibitor NC + si-CROCC group (*P* < 0.05). The inhibitor NC group and the miR-33b inhibitor + si-CROCC group had similar EdU positive expression rate and cell viability, as compared with the inhibitor NC + si-NC group (*P* > 0.05). The aforementioned findings suggested that miR-33b results in CROCC down-regulation, thus impeding the cell proliferation of GBC.

### MiR-33b down-regulates the expression of CROCC to inhibit migration and invasion in GBC cells

**Figure 4:**
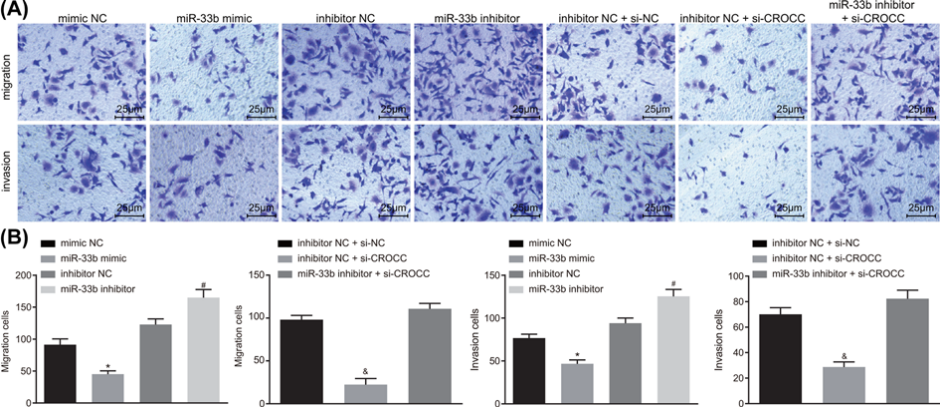
MiR-33b down-regulates the expression of CROCC to inhibit cell migration and invasion in GBC cells (**A**) Representative images of cell migration and invasion of cells in each group as detected by Transwell assay. (**B**) Quantitative analysis for cell migration and invasion in each group; *, *P* < 0.05 vs. the mimic NC group, #, vs. the inhibitor NC group; &, vs. the inhibitor NC + si-NC group; the data in the figure were analyzed by one-way ANOVA, and the experiment was repeated three times independently; miR-33b, microRNA-33b, NC, negative control, GBC, gallbladder carcinoma.

Transwell assay was conducted to illustrate the effects of miR-33b on migration and invasion in GBC cells. The results (Figure 4A,B) indicated that in comparison with the mimic NC group, the miR-33b mimic group exhibited significantly reduced cell migration and invasion (*P* < 0.05). In comparison with the inhibitor NC group, cell migration and invasion were prominently elevated in the miR-33b inhibitor group (*P* < 0.05). In contrast with the inhibitor NC + si-NC group, the inhibitor NC + si-CROCC group showed apparent descended cell migration and invasion (*P* < 0.05). No significant difference in cell migration and invasion was observed in the miR-33b inhibitor + si-CROCC group in comparison with the inhibitor NC + si-NC group (*P >* 0.05). These results demonstrated that the miR-33b down-regulates CROCC to inhibit cell migration and invasion in GBC.

### MiR-33b down-regulates CROCC expression to inhibit EMT in GBC cells

**Figure 5:**
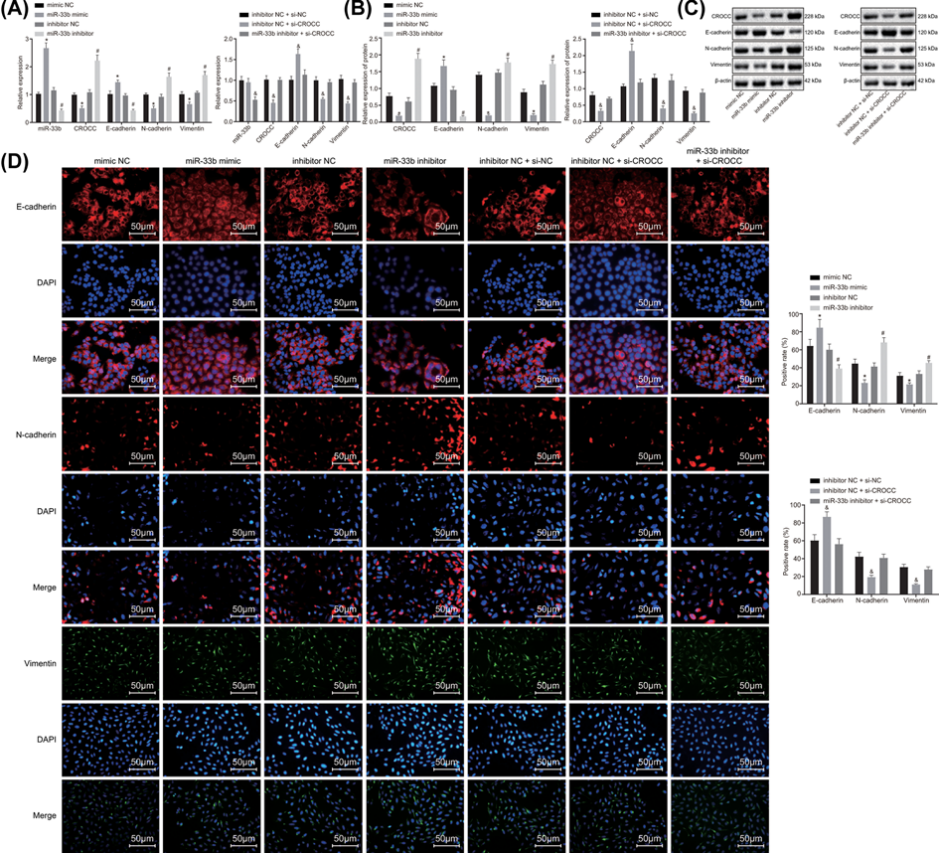
MiR-33b inhibits the EMT in GBC cells by down-regulating CROCC expression (**A**) Expression of miR-33b, CROCC, E-cadherin, N-cadherin, and Vimentin as detected by RT-qPCR. (**B**) Protein levels of E-cadherin, CROCC, N-cadherin, and Vimentin as detected by Western blot analysis. (**C**) The protein band map of E-cadherin, CROCC, N-cadherin, and Vimentin as detected by Western blot analysis. (**D**) Representative images and quantification of expression of E-cadherin, CROCC, N-cadherin, and Vimentin as detected by immunofluorescence staining; *, *P* < 0.05 vs. the mimic NC group, #, vs. the inhibitor NC group, &, vs. the inhibitor NC + si-NC group; the data in the figure were analyzed by one-way ANOVA, and the experiment was repeated three times independently; RT-qPCR, reverse transcription quantitative polymerase chain reaction; miR-33b, microRNA-33b, NC, negative control.

We performed RT-qPCR and Western blot analysis (Figure 5A–C) to evaluate the levels of CROCC, E-cadherin, N-cadherin, and Vimentin. The results showed that compared with the mimic NC group, the miR-33b mimic group presented with significantly elevated levels of miR-33b (*P* < 0.05) and E-cadherin but reduced levels of CROCC, N-cadherin, and Vimentin (*P* < 0.05). In comparison with the levels observed in the inhibitor NC group, the levels of miR-33b and E-cadherin declined (*P* < 0.05) while the mRNA and protein levels of CROCC, N-cadherin, and Vimentin had elevated (*P* < 0.05) in the miR-33b inhibitor group. In comparison with the inhibitor NC + si-NC group, no significant difference was evident in the inhibitor NC + si-CROCC group (*P* > 0.05) while the levels of CROCC, N-cadherin and Vimentin obviously declined, while the expression of E-cadherin increased (*P* < 0.05). A cohesive comparison of the inhibitor NC group and the inhibitor NC + si-NC group revealed that the miR-33b inhibitor + si-CROCC group had decreased expression profiles of miR-33b and CROCC (*P* < 0.05), while the mRNA and protein levels of E-cadherin, N-cadherin, and Vimentin showed no significant difference (*P* > 0.05).

Immunofluorescence was conducted to detect the expression of EMT-related markers. The results indicated (Figure 5D) that E-cadherin was expressed in cell cytoplasm with predominant expression in the membrane. N-cadherin was expressed in the cell membrane and primarily in the cytoplasm. Vimentin was expressed in intercellular substance. In comparison with the mimic NC group, the miR-33b mimic group showed elevated expression of E-cadherin (*P* < 0.05) along with decreased levels of N-cadherin and Vimentin (*P* < 0.05). On comparing with the inhibitor NC group, the level of E-cadherin declined (*P* < 0.05) while the levels of N-cadherin and Vimentin increased in the miR-33b inhibitor group (*P* < 0.05). In comparison with the inhibitor NC + si-NC group, the inhibitor NC + si-CROCC group presented with an increased expression of E-cadherin while diminished levels of N-cadherin and Vimentin (*P* < 0.05). No obvious difference was observed in the levels of E-cadherin, N-cadherin, and Vimentin in the miR-33b inhibitor + si-CROCC group, in comparison with those observed in the inhibitor NC + si-NC group (*P* > 0.05). These findings concluded that miR-33b could effectively suppress EMT in GBC by down-regulating CROCC.

### MiR-33b down-regulates the expression of CROCC to inhibit tumor growth and EMT in GBC *in vivo*

**Figure 6:**
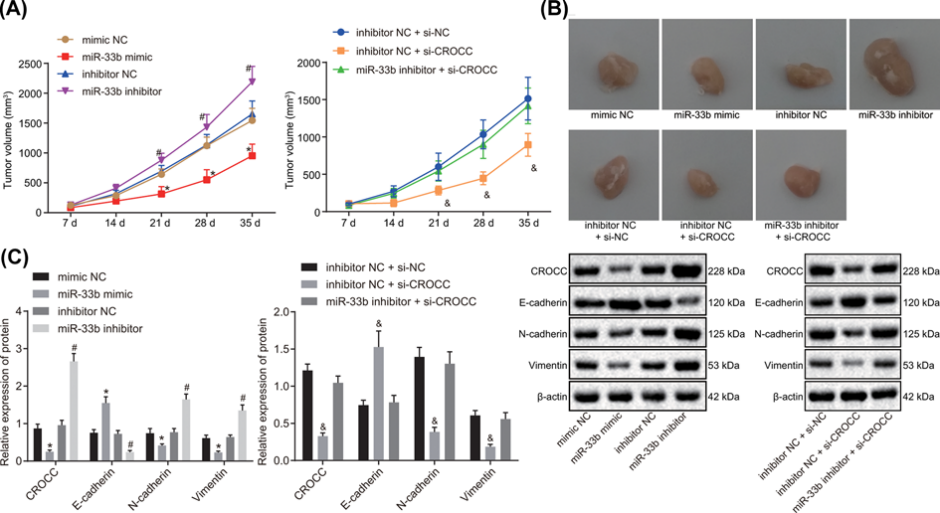
MiR-33b inhibited tumor growth and EMT in nude mice treated with GBC cells by down-regulating CROCC *in vivo* (**A**) Tumor volume growth rate with time in each group. (**B**) Observation of tumor size in each group after cell transfection. (**C**) Protein expression and bands of CROCC and EMT-related proteins in GBC tissues of nude mice as detected by Western blot analysis. *, *P* < 0.05 vs. the mimic NC group, #, vs. the inhibitor NC group, &, vs. the inhibitor NC + si-NC group; the data in the figure among multiple groups were analyzed by one-way ANOVA, and data among multiple groups at different time points were analyzed by repeated measures ANOVA; the experiment was repeated three times independently; miR-33b, microRNA-33b; NC, negative control; GBC, gallbladder carcinoma.

Transfected cells were injected into nude mice to verify the effects of miR-33b on GBC tumor growth *in vivo*. The results (Figure 6A,B) suggested that compared with the mimic group, the tumor growth rate and the tumor size in nude mice decreased in the miR-33b mimic group (*P* < 0.05). Tumor growth rate was the fastest with an augmented tumor size (*P* < 0.05) in the miR-33b inhibitor group compared with the inhibitor NC group. In comparison with the inhibitor NC + si-NC group, the tumor growth rate and tumor size were observed to be decreased in the inhibitor NC + si-CROCC group (*P* < 0.05). No evident difference in tumor growth rate was illustrated between the inhibitor NC group and the miR-33b inhibitor + si-CROCC group (*P >* 0.05). The findings suggested that miR-33b could inhibit the tumor growth in nude mice with GBC through down-regulating CROCC.

Moreover, the expression of the proteins extracted from subcutaneous tumors of the nude mice was measured by conducting Western blot analysis. The results (Figure 6C) showed that compared with the mimic NC group, the miR-33b mimic group presented with an elevated expression of E-cadherin and reduced expression of CROCC, N-cadherin, and Vimentin. In comparison with the inhibitor NC group, the expression of E-cadherin was down-regulated while that of CROCC, N-cadherin, and Vimentin was up-regulated in the miR-33b inhibitor group. Additionally, in contrast with the inhibitor NC + si-NC group, the inhibitor NC + si-CROCC group exhibited an increased expression of E-cadherin but decreased expressions of CROCC, N-cadherin, and Vimentin. These findings suggest that miR-33b inhibits EMT of GBC by negatively regulating CROCC.

## Discussion

GBC is a relatively rare form of malignancy but with a high mortality rate [18]. The aberrant expression of certain miRs has been demonstrated to be functionally involved in GBC progression through regulation of various cellular processes such as cell proliferation, migration, invasion, and apoptosis [19]. Herein, our study investigated the effect of miR-33b on the regulation of EMT in GBC cells. Consequently, our findings illustrated that up-regulation of miR-33b could potentially suppress cell proliferation, migration, invasion, and EMT in GBC through down-regulation of CROCC.

In the initial experiment, the GBC cell lines presented with down-regulated expression of miR-33b. Furthermore, reports have documented miR-33b to be down-regulated in patients with high-risk genetic abnormality and associates with poor prognosis in multiple myeloma (MM) [20]. Interestingly, Tian et al. also flagged the presence of a low expression of miR-33b in MM, which confirmed the functionality of miR-33b as a tumor-suppressor in MM cells [21]. Therefore, miR-33b was speculated to serve as a prognostic marker for cancer progression [22]. Similarly, a recent study revealed that miR-125b-5p was down-regulated in GBC [23]. The findings from following experiments illustrated CROCC as a target gene of miR-33b, with miR-33b accounting for significantly elevated expression of E-cadherin but decreased levels of CROCC, N-cadherin, and Vimentin. These findings were in consistency with the result of dual luciferase report gene assay. Studies have demonstrated the vital functionality of EMT during cancer development as well as in GBC progression, with its deregulation facilitating tumor metastasis [24,25]. E-cadherin and Vimentin are considered as principal biomarkers of EMT; however, E-cadherin is commonly disoriented along the progression of EMT [26]. A former study demonstrated a correlation of loss of E-cadherin expression with promoted invasive capacity and high tumor grade and poor prognosis [27]. Moreover, restoration of miR-33b expression inhibited lung adenocarcinoma cell proliferation, migration, and invasion and tumor cell EMT *in vitro*, which conformed to a previous study supporting the functionality of an altered miR expression to be vital in cancer metastasis [28]. Besides, a recent research observed similar results demonstrating that the overexpression of miR-30a enhanced E-cadherin expression but reduced the levels of N-cadherin and Vimentin [29].

Our study also demonstrated that miR-33b could suppress various cellular processes such as invasion, migration, proliferation, EMT, and tumor growth of GBC through down-regulating the expression of CROCC. In consistency with our study, Wu et al. also observed that the overexpression of miR-33b suppressed the proliferation, migration, and invasion of hepatocellular carcinoma cells [30]. Also, an enforced expression of miR-33b resulted in its inhibitory effects on EMT in lung cancer cells [31]. Furthermore, a recent study also revealed the potential of miR-33b to serve as an anti-metastatic miR, with overexpressed miR-33b greatly inhibiting cell proliferation in lung squamous cell cancer [10]. In consistency with our results, reports have observed miR-33b to inhibit tumor growth and metastasis of breast cancer cells *in vivo* [32]. CROCC has also been demonstrated to be of critical functionality with dual motives in regulating both tumorigenicity and aberrant centrosome duplication [33]. In addition, the miR-33b has also been identified to function as a suppressive miR in breast cancer progression by inhibiting the stemness and metastasis of breast cancer cells [32]. A current study suggested that overexpression of miR-33b due to its tumor suppressive properties inhibited osteosarcoma cell migration and invasion by targeting the c-Myc gene [11]. Moreover, overexpression of miR-1 and miR-145 could potentially inhibit GBC cell growth *in vitro* by decreasing cell proliferation and suppressing colony formation or clonogenic survival, which was in accordance with our results [34].

To conclude, our findings demonstrated that miR-33b could inhibit GBC proliferation, migration, invasion, and EMT through down-regulating CROCC. Our findings aid in the speculation of miR-33b to serve as a cancer suppressor in GBC and a potential therapeutic target for GBC treatment. In future studies, a larger vivo experiment should be conducted to further determine the underlying mechanism of miR-33b in GBC.

## Abbreviations

ANOVA: analysis of variance
CROCC: ciliary rootlet coiled coil protein
GBC: gallbladder cancer
HRP: horseradish peroxidase
HSRRB: Health Research Resource Bank
miR-33b: microRNA-33b
MUT: mutant type
PBS: phosphate-buffered saline
RT-qPCR: reverse transcription quantitative polymerase chain reaction
SERBP-1: sterol response element binding protein 1
TAX1BP2: TAX1 binding protein 2
TBST: tris-buffered saline tween
WT: wild-type
