# Expression of Concern: Up-regulated microRNA-33b inhibits epithelial-mesenchymal transition in gallbladder cancer through downregulating CROCC

**DOI:** 10.1042/BSR-2019-0108_EOC

**Published:** 2024-11-15

**Keywords:** CROCC, Epithelial-mesenchymal transition, Gallbladder cancer, microRNA-33b, Proliferation

The Editorial Office has been made aware of potential issues surrounding the scientific validity of this paper “Up-regulated microRNA-33b inhibits epithelial–mesenchymal transition in gallbladder cancer through down-regulating CROCC” 10.1042/BSR20190108, hence has issued an expression of concern to notify readers. It has been noted that the Western blots seem to resemble the appearance of those in other publications by different authors, and the acknowledgements may not be relevant to the published article. The authors were contacted regarding the concerns, however so far the Editorial Office has not received a response.

